# Degrees and Diplomas

**Published:** 1909-05-01

**Authors:** Frederick w. Collingwood

**Affiliations:** Dewhurst, Acton Hill, W.


### Editor's Letter-Box.

### DEGREES AND DIPLOMAS.

*To the Editor of* The Hospital.

Sir,—It is doubtful whether State examinations would have those beneficial results anticipated by the sanguine. At the present time an amount of uniformity is attained by the jurisdiction and inspection of the General Medical Council. If it is thought by this body that the general standard of medical examinations should be raised, it is within its powers to bring about this result, and remedial measures are insured from time to time by resolutions of this body.

I further submit that the competition between universities and universities and corporations and universities at the present day produces well-thought-out courses of study and efficient teaching, and that slight variations in courses of study, etc., tend on comparison to produce alterations towards further efficiency. One question, to my mind, is whether, if the present standard of the London University had been and were maintained as the standard of a university degree all round, it would not have excluded many men of genius from the higher ranks of the medical profession; and whether many men at the present time recognised as persons of light and learning would have ever been able to shine had the said standard been universal (for men without a degree are practically excluded from many posts of honour in the profession). If this is so, is not the present standard unnecessarily severe? A stereotyped State examination, on the other hand, would probably require a very mediocre standard, and the importance attached to such a Government examination would tend to put into the shade all others, and absence of healthy competition would tend to lower rather than raise the standard of medical examination requirements. The Legislature may be useful to remedy defects in existing bodies, but if the Government take upon themselves to conduct examinations, what body is there left to remedy defects in Governmental examinations? Government would become judge in its own cause. But there seems no reason why Londoners should not have an attainable Degree.

Yours faithfully,

Frederick W. Collingwood.

Dewhurst, Acton Hill, W., April 16.

